# Gut Microbiota Shifts After a Weight Loss Program in Adults with Obesity: The WLM3P Study

**DOI:** 10.3390/nu17142360

**Published:** 2025-07-18

**Authors:** Vanessa Pereira, Amanda Cuevas-Sierra, Victor de la O, Rita Salvado, Inês Barreiros-Mota, Inês Castela, Alexandra Camelo, Inês Brandão, Christophe Espírito Santo, Ana Faria, Conceição Calhau, Marta P. Silvestre, André Moreira-Rosário

**Affiliations:** 1 NOVA Medical School, Faculty of Medical Sciences, NMS, FCM, Nova University of Lisbon, 1169-056 Lisbon, Portugal; ritasalvado@usal.es (R.S.); ines.mota@nms.unl.pt (I.B.-M.); ines.castela@nms.unl.pt (I.C.); ana.faria@nms.unl.pt (A.F.); ccalhau@nms.unl.pt (C.C.); marta.silvestre@nms.unl.pt (M.P.S.); andre.rosario@nms.unl.pt (A.M.-R.); 2 Nutrition Department Farmodiética, Farmodiética, 2785-723 Lisbon, Portugal; 3 Precision Nutrition and Cardiometabolic Health, IMDEA-Alimentación Institute (Madrid Institute for Advanced Studies), Campus of International Excellence (CEI), Universidad Autónoma de Madrid (UAM), Spanish National Research Council (Consejo Superior de Investigaciones Científicas, CSIC), 28049 Madrid, Spain; victor.delao@alimentacion.imdea.org; 4 Faculty of Health Sciences, International University of La Rioja (UNIR), 26006 Logroño, Spain; 5 Primary Care Research Unit of Salamanca (APISAL), Salamanca Primary Healthcare Management, Castilla y León Regional Health Authority (SACyL), Institute of Biomedical Research of Salamanca (IBSAL), 37007 Salamanca, Spain; 6 Comprehensive Health Research Center (CHRC), NOVA Medical School, Faculty of Medical Sciences, NMS, FCM, NOVA University Lisbon, 1169-056 Lisbon, Portugal; 7 CATAA—Agro-Food Technological Support Center, Industrial Zone of Castelo Branco, Rua A, 6000-459 Castelo Branco, Portugal; alexandra.camelo@cataa.pt (A.C.); inesbrandao@cataa.pt (I.B.); cespiritosanto@cataa.pt (C.E.S.); 8 Center for Functional Ecology Science for People & the Planet, TERRA Associated Laboratory, Department of Life Sciences, University of Coimbra, Calçada Martim de Freitas, 3000-456 Coimbra, Portugal; 9 CINTESIS@RISE, NOVA Medical School, Faculty of Medical Sciences, NMS, FCM, NOVA University Lisbon, 1169-056 Lisbon, Portugal

**Keywords:** dietary interventions, gut microbiota, *Faecalibacterium*, weight loss, fat mass loss, visceral fat loss

## Abstract

Background: The gut microbiota is increasingly recognized as a key modulator in obesity management, influencing host energy balance, lipid metabolism, and inflammatory pathways. With obesity prevalence continuing to rise globally, dietary interventions that promote beneficial microbial shifts are essential for enhancing weight loss outcomes and long-term health. Objective: This study investigated the effects of the multicomponent Weight Loss Maintenance 3 Phases Program (WLM3P), which integrates caloric restriction, a high-protein low-carbohydrate diet, time-restricted eating (10h TRE), dietary supplementation (prebiotics and phytochemicals), and digital app-based support on gut microbiota composition compared to a standard low-carbohydrate diet (LCD) in adults with obesity. The analysis focused exclusively on the 6-month weight loss period corresponding to Phases 1 and 2 of the WLM3P intervention. Methods: In this sub-analysis of a randomized controlled trial (ClinicalTrials.gov Identifier: NCT04192357), 58 adults with obesity (BMI 30.0–39.9 kg/m^2^) were randomized to the WLM3P (*n* = 29) or LCD (*n* = 29) groups. Stool samples were collected at baseline and 6 months for 16S rRNA sequencing. Alpha and beta diversity were assessed, and genus-level differential abundance was determined using EdgeR and LEfSe. Associations between microbial taxa and clinical outcomes were evaluated using regression models. Results: After 6-month, the WLM3P group showed a significant increase in alpha diversity (*p* = 0.03) and a significant change in beta diversity (*p* < 0.01), while no significant changes were observed in the LCD group. Differential abundance analysis revealed specific microbial signatures in WLM3P participants, including increased levels of *Faecalibacterium*. Notably, higher *Faecalibacterium* abundance was associated with greater reductions in fat mass (kg, %) and visceral adiposity (cm^2^) in the WLM3P group compared to LCD (*p* < 0.01). Conclusions: These findings suggest a potential microbiota-mediated mechanism in weight loss, where *Faecalibacterium* may enhance fat reduction effectiveness in the context of the WLM3P intervention.

## 1. Introduction

Obesity has reached epidemic proportions globally [1], demanding innovative and sustainable treatment strategies beyond conventional caloric restriction and lifestyle modification, which often fail to produce lasting results due to the complex and heterogeneous nature of obesity [2,3].

In this context, the gut microbiota has emerged as a promising modifiable target for obesity management, as it plays a central role in regulating energy harvest, lipid metabolism, glycemic control, and low-grade inflammation [4,5,6]. Obesity-related dysbiosis typically involves reduced microbial diversity and depletion of beneficial short-chain fatty acid (SCFA)-producing taxa such as *Faecalibacterium*, *Christensenellaceae*, and *Akkermansia*, contributing to metabolic dysfunction and impaired gut barrier integrity [4,5,6,7].

Among these taxa, *Faecalibacterium* has emerged as a promising microbial marker of dietary responsiveness, consistently linked to reductions in fat mass, enhanced insulin sensitivity, and improved inflammatory profiles during weight loss interventions [5,7,8,9,10]. Other taxa, such as *Christensenellaceae R-7* group, have similarly been linked to leanness and beneficial metabolic traits [4,5,6,11].

Diverse dietary interventions can reshape the gut microbiota, often enhancing SCFA-producing taxa while reducing pro-inflammatory genera [4,5,6]. For instance, caloric restriction is known to remodel the gut microbiota by reducing substrate availability for proteolytic fermentation while favoring taxa associated with improved energy efficiency and metabolic health, including *Akkermansia* and *Christensenellaceae,* although human responses are variable [5,12,13,14]. High-fiber diets increase *Faecalibacterium* and *Bifidobacterium*, improving metabolic and inflammatory markers [15,16]. Low-carb and ketogenic diets tend to enrich *Bacteroides* and *Akkermansia*, though they may reduce microbial diversity [17,18]. High-protein diets can increase proteolytic bacteria such as *Bacteroides* [9] but may reduce beneficial SCFA producers if fiber intake is low [19]. Time-restricted eating (TRE), an increasingly popular form of intermittent fasting, has been shown to modulate gut microbiota by aligning microbial rhythms with host circadian cycles, increasing alpha diversity and enriching butyrate-producing taxa such as *Faecalibacterium*, while reducing endotoxemia-associated microbes [20,21]. Given the multifactorial nature of obesity, focusing on isolated dietary strategies may offer limited benefits in achieving meaningful and sustained microbiota remodeling and metabolic improvements [4,5,6].

Furthermore, most studies assess only pre–post intervention comparisons, failing to distinguish microbial dynamics occurring specifically during the active weight loss phase, when physiological shifts are most pronounced [12,13,14]. Given the rapid responsiveness of gut microbiota to dietary changes, isolating the initial phase of weight loss provides a unique window to capture early microbial adaptations that may influence, or mirror shifts in adiposity and metabolic health, offering insights into potential microbial biomarkers of dietary responsiveness.

To address these gaps, the present study investigates gut microbiota dynamics specifically during the 6-month active weight loss phase of the Weight Loss Maintenance 3 Phases program (WLM3P) [22], a multicomponent intervention combining caloric restriction, a high-protein, low-carbohydrate diet, 10-h TRE, targeted supplementation, and app-based behavioural support. We compare gut microbial shifts and associated metabolic outcomes with those of a standard low-carbohydrate diet (LCD), focusing on microbial diversity, taxonomic changes, and correlations with body composition and lipid markers. By identifying specific microbial changes that correlate with improvements in fat mass and visceral adiposity, we aim to provide a rationale for future clinical trials that incorporate targeted prebiotics or microbial consortia to enhance weight loss efficacy. Ultimately, this research may inform the development of personalized dietary strategies that optimize gut microbiota composition to promote sustainable weight loss and improve metabolic health.

## 2. Materials and Methods

### 2.1. Trial Design

This is a sub-study of the WLM3P study [22], a randomized controlled trial conducted in adults (aged 18–65 years) with obesity (BMI: 30–40 kg/m^2^), comprising a total of 18 months (6-month weight loss period followed by a 12-month weight maintenance period) [22]. However, this sub-study focuses specifically on the gut microbiota dynamics during the initial 6-month weight loss period of the WLM3P trial. This investigation was conducted at NOVA Medical School, NOVA University of Lisbon, between March 2020 and January 2023. The intervention group (WLM3P) underwent a multicomponent intervention (7 components), and the control group (LCD) underwent a standard low carb diet. All participants provided written informed consent in accordance with the principles of the Declaration of Helsinki [22]. The study was approved by the Ethics Committee of the NOVA Medical School (Lisbon, Portugal) (CEFCM Approval Number: 108/2018) and registered at www.clinicaltrials.gov (NCT04192357) before participants’ recruitment. Exclusion and inclusion criteria have been published previously [22].

### 2.2. Participants

For this analysis, a sub-sample of 58 participants was included [WLM3P (*n* = 29) and LCD (*n* = 29)]. All participants adhered to the protocol, providing fecal samples for microbiota analysis as instructed and signing informed consent forms.

### 2.3. Dietary Interventions

To induce WL, a personalized dietary plan was prescribed for each participant, aiming for 70% of their daily energy requirements (DER) in both dietary interventions (baseline to month 6). The DER was estimated by multiplying the basal metabolism rate provided by the body composition analyser (Inbody^®^ model 770; InBody, Seoul, Republic of Korea) by the individual’s physical activity level (estimated using the International Physical Activity Questionnaire; IPAQ). In both dietary interventions, individual preferences were respected, and the culinary methods were based on the Mediterranean diet [23]. Participants were provided with detailed plans containing allowed and not-allowed foods and recipes to promote healthy food choices, which were designed to have the designed macronutrient profile.

#### 2.3.1. WLM3P (Intervention Group)

The WLM3P is a structured nutritional and behavioral program based on seven components: Dietary intervention with 3 Phases (Phase 1 and Phase 2—weight loss period, and Phase 3—weight maintenance period), regular one-to-one consultations, behavioral strategies, time-restricted eating (10-h TRE), dietary supplements, high-protein specific food, and a mobile and web application. For the purposes of this study, we focused exclusively on the weight loss period encompassing Phase 1 and Phase 2 of the WLM3P intervention. Phase 1 and Phase 2 consisted of approximately 10–20%, 35–40%, and 40–45% of total energy from carbohydrates, fats, and proteins, emphasizing the consumption of unsaturated fatty acids, high-quality carbohydrates including whole grains, fresh vegetables, and low glycemic index fruits. The 10-h TRE required participants to consume the provided meals within a 10-h window each day. At 6 months, participants had one-to-one weekly consultations (24 presential sessions) when weight loss, biochemical data, and fecal samples were collected.

#### 2.3.2. LCD (Active Control Group)

Participants randomized to the active control group received a standard carbohydrate-restricted diet [22]. For 6 months, participants had 6 presential sessions one-to-one.

The LCD was designed to represent standard-of-care nutritional counselling, which typically includes fewer contact hours. While the WLM3P involved 24 one-on-one sessions due to its behavioural and educational components, the LCD group’s 6 sessions reflect common clinical practice, allowing comparison between intensive and standard interventions [2,3].

### 2.4. Body Measurements and Composition

Anthropometric measurements, including weight, waist and hip circumference, were collected at the beginning of the study by trained nutritionists using conventional validated procedures. Body mass index (BMI) was calculated as the ratio between body weight and squared height (kg/m^2^) and the BMI classification criteria was following according to the World Health Organization [24]. Body Composition was evaluated at baseline and after six months by a trained nutritionist using bioelectrical impedance analysis (Inbody^®^ model 770; InBody, Seoul, Republic of Korea). Body mass index (BMI) and waist circumference (WC) were measured according to the Directorate-General of Health’s guidelines [25].

#### 2.4.1. Biochemical Measurements

Venous blood samples were drawn by venipuncture after a 12 h overnight fast in a clinical setting. Blood tests were conducted with an automatized analyzer Pentra C200 and suitable kits were provided by the company (HORIBA Medical, Madrid, Spain). The following biochemical markers were assessed in the blood samples, assessing high-density lipoprotein cholesterol (HDL-c), triglycerides, and low-density lipoprotein cholesterol (LDL-c), following the instructions provided by the manufacturers.

#### 2.4.2. Fecal Sample Collection and Sequencing of Gut Microbiota

Regarding stool collection and microbiota analysis, participants used EasySampler^®^ Kits (ALPCO, Salem, NH, USA) to self-collect stool samples at home. Samples were promptly frozen at −80 °C, delivered to the study centres within 1–3 days, and stored until processing. The isolation of DNA from fecal samples was performed with the QIAamp^®^ DNA kit (Qiagen, Hilden, Germany) following the manufacturer’s protocol. DNA purity was measured using a NanoDrop spectrophotometer (Thermo Scientific; Waltham, MA, USA), and DNA concentration was quantified with a Qubit 4 Fluorometer (Thermo Scientific; Waltham, MA, USA). Libraries were prepared following the manufacturer’s protocol for 16S Barcoding Kit with minor modifications (SQK-16S024; Oxford Nanopore Technologies, ONT), gene amplification was performed using the LongAmp Hot Start Polymerase (New England Biolabs; Ipswich, MA, USA). Barcoded universal primer set (27F and 1492R) was employed to amplify the full length (1465 bp) of the bacterial 16S rRNA gene. A total of 24 samples were pooled at equimolar concentrations and loaded into a MinION flow cell (FLO-MIN106D R9.4.1, ONT) using a MinION MK1C sequencing device. Sequencing was conducted for 12–to 18 h and controlled via MinKNOW software (v21.11.7; ONT) for basecalling and quality checks, yielding between 1.93–2.95 million reads per run with an average read length of 1440 bp. Raw sequencing reads were processed using the BugSeq 16S pipeline, which performs quality filtering, adapter trimming, and clustering into Amplicon Sequence Variants (ASVs) using DADA2. Taxonomic classification was achieved using the SILVA v132 16S rRNA gene database, providing high-resolution assignments. Relative ASV abundances were calculated using the Phyloseq R package (version 2.5-7). To ensure data accuracy, read quality was monitored with NanoFilt, retaining only those reads with an average quality score above Q10 and a minimum length of 500 bp. ASV abundance matrices were filtered and normalized at taxonomic levels spanning ASV, species, genus, family, order, class, and phylum.

### 2.5. Statistical Analysis

Data were classified as parametric or nonparametric based on the Shapiro–Wilks and Smirnov–Kolmogorov test. For descriptive analysis, continuous nonparametric data were shown as median and interquartile range. To compare the differences in the unpaired groups, nonparametric tests were conducted. For all analyses, significance was determined as two t-tailed and *p*  <  0.05. Mixed-effects models were conducted to evaluate changes in anthropometric and biochemical variables between WLM3P and LCD groups across two time points: baseline and six months. The models included interaction terms for group and time to examine differential effects over time between groups. Specifically, we evaluated between-group differences at baseline and six months, within-group changes over time, and the interaction effect reflecting the differential change between groups from baseline to six months. Results were reported as mean differences with 95% confidence intervals. Gut bacterial diversity was evaluated by Shannon index and gut bacterial richness was measured by Chao1 index. Beta diversity was assessed using the Bray–Curtis dissimilarity to evaluate differences in the composition of the global microbial community between groups. The distances (or dissimilarity) between samples of the same group were compared to the distances between groups using Permutational Multivariate Analysis of Variance (PERMANOVA). The *p* value was calculated through the Pairwise PERMANOVA method to compare β diversity between each category in all samples. Given the paired structure of the data (baseline vs. 6-month samples), the Wilcoxon signed-rank test was applied for within-group comparisons. The Benjamini-Hochberg false discovery rate (FDR) procedure to control for type I error in the context of multiple hypothesis testing.

Linear discriminant analysis (LDA) effect size (LEfSe) was used to explore microbial taxa with differential abundance between different study periods and groups, (version 1.0). Taxa showing LDA values above 2.0 at a value of *p* < 0.05 were considered enriched taxa in each group. Edge R analysis was used for the comparison between groups and adjusting by FDR. Data were normalized by the centered-log ratio (CLR). Heatmaps displaying Spearman pairwise correlations were generated using the ComplexHeatmap package, highlighting only correlations with *p*-values < 0.05, corrected by FDR. Potential interactions between bacterial variables and fat mass loss (%) were investigated with general linear regression models that introduced the corresponding interaction terms into the models, which were adjusted for age, sex and BMI using Stata 12. (StataCorp LLC, College Station, TX, USA).

## 3. Results

### 3.1. Baseline Data and Effect of the WLMP3P Intervention Compared with Control Group on the Main Anthropometric and Biochemical Variables After 6 Months: Mixed-Effects Model Results

Baseline demographic characteristics were comparable between the WLM3P intervention group (mean age: 43 ± 8 years; 34.5% male) and the LCD control group (mean age: 47 ± 9 years; 30.0% male), with no significant differences in age (*p* = 0.907) or gender distribution (*p* = 0.897). Longitudinal changes in anthropometric and biochemical parameters were analyzed using mixed-effects models with fixed effects for time, intervention group, and their interaction, along with random effects to account for intra-individual variability (Table 1). At six months, the WLM3P intervention demonstrated superior efficacy compared to LCD in reducing: weight (Δ = −6.50 kg, 95% CI: −11.93 to −1.07; *p* = 0.01), BMI (Δ = −2.61 kg/m^2^, 95% CI: −3.69 to −1.52; *p* < 0.001), fat mass (Δ = −6.09 kg, 95% CI: −9.57 to −2.61; *p* < 0.001), fat mass percentage (Δ = −5.06%, 95% CI: −8.70 to −1.42; *p* < 0.001), visceral fat area (Δ = −30.70 cm^2^, 95% CI: −49.78 to −11.61; *p* < 0.001), waist circumference (Δ = −8.93 cm, 95% CI: −13.34 to −4.52; *p* < 0.001) and waist-to-hip ratio (Δ = −0.04, 95% CI: −0.07 to −0.01; *p* = 0.02). All parameters showed significant time-by-intervention interactions (*p* < 0.001), indicating a more pronounced effect of WLM3P over the 6-month period. These results suggest that WLM3P is more effective than LCD in improving body composition and anthropometric measures associated with obesity. The between-group differences in muscle mass reduction, LDL-cholesterol, HDL-cholesterol, triglycerides and Tg/HDL ratio were not statistically significant (Table 1).

### 3.2. Comparison of Gut Microbiota Composition Between WLM3P and LCD Groups

**Figure 1:**
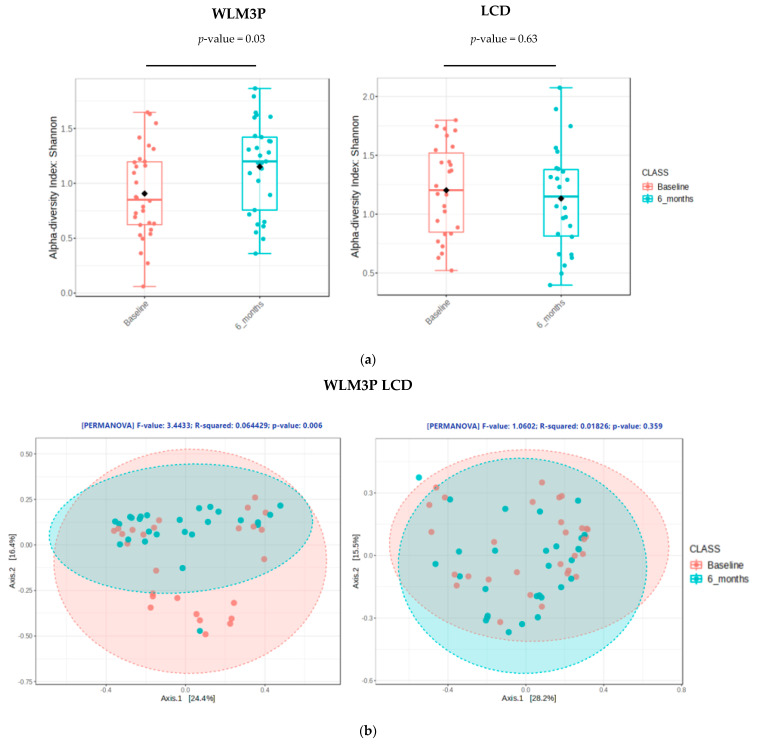
(**a**) Alpha diversity analysis evaluated by Shannon index at baseline and after 6 months of intervention, by groups (WLM3P and LCD). Pink boxes represent Shannon index values at baseline and blue boxes represent Shannon index after 6 months, compared by Wilcoxon test. (**b**) Principal coordinates analysis (PCoA) of beta diversity changes in WLM3P group and LCD using Bray−Curtis distances and compared by PERMANOVA test. Pink dots represent values at baseline and blue dots represent values after 6 months.

Alpha diversity metrics were used to measure species richness and evenness in the groups. The Shannon Index (Figure 1a) was calculated and revealed significantly higher values for the WLM3P group after 6 months of intervention (*p* = 0.03). However, no significant results were found in alpha diversity after 6 months in LCD group (*p* = 0.63).

Beta diversity metrics were used to compare differences in the community composition in each group of the population (Figure 1b). Beta diversity was significantly different in WLP3P group after 6 months of intervention (*p* < 0.01). No statistically significant differences in microbial diversity within LCD group at the end of study (*p* = 0.36).

### 3.3. Analysis of the Differential Microbiota Abundances at Genus Level Between WLM3P and LCD Groups After 6 Months of Intervention

Table 2 and Table 3 present the bacterial genus that presented a significant difference in relative abundance for WLM3P group (Table 2) and LCD (Table 3) after 6 months, compared by EdgeR. The analysis of genus abundance in the WLM3P group after 6 months revealed significant changes in gut microbiota composition. There was a significant decrease in the abundance of *Romboutsia* (FDR = 5.25 × 10^−7^), *Family XIII AD3011 group* (FDR = 2.63 × 10^−6^), and *Subdoligranulum* (FDR = 3.90 × 10^−4^). In contrast, there was a significant increase in the abundance of genera previously associated with metabolic improvements such as *Phascolarctobacterium* (FDR = 5.8 × 10^−4^) and *Christensenellaceae_R_7_group* (FDR = 0.002). In the LCD group, significant increases were observed in the abundance of *Bacteroides* (FDR = 0.001), *Lachnospira* (FDR = 0.003), *Phascolarctobacterium* (FDR = 0.003), *Ruminococcaceae_UCG_014* (FDR = 0.013), *Ruminococcaceae_UCG_005* (FDR = 0.017) and *Ruminococcaceae_UCG_002* (FDR = 0.048), suggesting a different yet significant impact on the gut microbiota compared to the WLM3P group.

**Figure 2:**
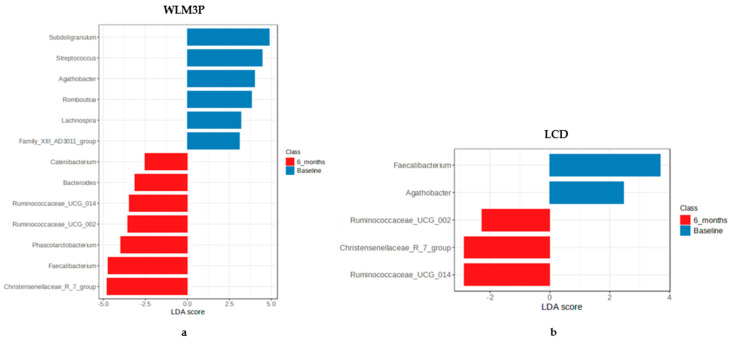
LEfSe analysis of microbial communities for WLM3P group (**a**) and for LCD group (**b**), by time. The figure illustrates the results of the Linear discriminant analysis Effect Size (LDA) applied to microbial communities. The bars represent the logarithmic LDA scores, indicating the effect size of different taxa across the experimental groups. Taxa with an LDA score greater than 2.0 are highlighted, suggesting significant differences in abundance. Blue colour represents the microbiota at baseline and red corresponds to microbiota composition after 6 months, providing a clear visual representation of the taxa that are significantly enriched or depleted in each group, by time.

In addition, a LEfSe analysis revealed significant differences in the microbial community composition between groups (WLM3P and LCD). Figure 2a shows an overrepresentation of *Subdoligranulum*, *Streptococcus*, *Agathobacter*, *Romboutsia* and *Lachnospira* genera at baseline in the WLM3P group. After 6 months, a total of 7 taxa (*Christensenellaceae R-7 group*, *Faecalibacterium*, *Phascolarctobacterium, Ruminococcaceae UCG_002*, *Ruminococcaceae UCG_014, Bacteroides* and *Catenibacterium*) were overrepresented in this group. In the LCD group (Figure 2b), *Faecalibacterium* and *Agathobacter* were significantly overrepresented at baseline. Conversely, after 6 months, *Ruminococcaceae_UCG-002*, *Ruminococcaceae_UCG-014* and *Christensenellaceae R-7* were overrepresented in this group.

### 3.4. Association Analysis Between Gut Microbiota and Anthropometric and Biochemical Changes After 6 Months in WLM3P and LCD Groups

**Figure 3:**
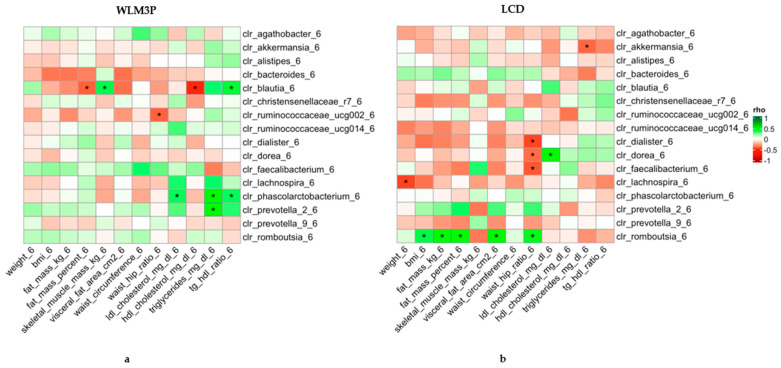
Heatmap of Spearman’s correlation coefficients between anthropometric and biochemical determinations and the abundance of microbial genera normalized by CLR after six months of intervention. The left panel (**a**) corresponds to the WLM3P group, and the right panel (**b**) to the LCD group. Each cell represents the correlation between a clinical parameter (x-axis) and a microbial genus (y-axis), with the color indicating the strength and direction of the correlation (green for positive, red for negative). Significant correlations are marked with an asterisk (*). The strength of the correlations (Spearman’s rho) is color-coded according to the scale on the right, ranging from −1 to 1.

Spearman pairwise correlation analyses were conducted with CLR adjustment to assess the gut microbiota composition at 6 months, as well as the changes in anthropometric and biochemical variables across both study groups, which are illustrated in Figure 3. The heatmaps display the Spearman’s correlations between anthropometric and biochemical determinations and the relative abundances of specific microbial genera after six months of intervention depending on the group of intervention (WLM3P and LCD).

In the WLM3P group (Figure 3a), significant positive correlations were observed between *Blautia* and tg/HDL ratio and with skeletal muscle after 6 months of intervention. *Phascolarctobacterium* showed a significant positive correlation with LDL cholesterol, triglycerides and tg/HDL ratio. Similarly, *Prevotella_2* presented a significant positive correlation with triglycerides in the same timeframe. In addition, *Blautia* presented a significant negative correlation with fat mass percentage and HDL cholesterol. Also, *Ruminococcaceae_ucg002* showed a significant negative correlation with waist hip ratio in this group after 6 months of intervention. On the other hand, in the LCD group (Figure 3b) *Akkermansia_6* showed a significant negative correlation with triglycerides. The waist-hip ratio after 6 months presented a significant negative correlation with *Dialister*, Dorea and *Faecalibacterium*. Also, Dorea showed a positive correlation with LDL cholesterol at 6 months. In addition, *Lachnospira* presented a significant negative correlation with weight at 6 months in this group of study. *Romboutsia* genus showed significant positive correlations with BMI, fat mass (Kg), fat mass percentage, visceral fat and waist-hip ratio at 6 months in LCD group.

### 3.5. Linear Regression Analysis for the Relationship Between Gut Microbiota Composition and Body Composition Variables in Each Group of Study

To explore the interaction between microbiota composition and the anthropometric and body composition differences observed between the WLM3P and LCD groups (Table 1), linear regression models with an interaction term were applied. These models assessed the impact of specific bacterial genera, particularly *Faecalibacterium*, on weight-related outcomes in each dietary intervention group. While several bacterial genera were tested (Table 2 and Table 3, Figure 2 and Figure 3), *Faecalibacterium* was the only genus that exhibited a significant interaction effect on anthropometric and body composition variables.

**Figure 4:**
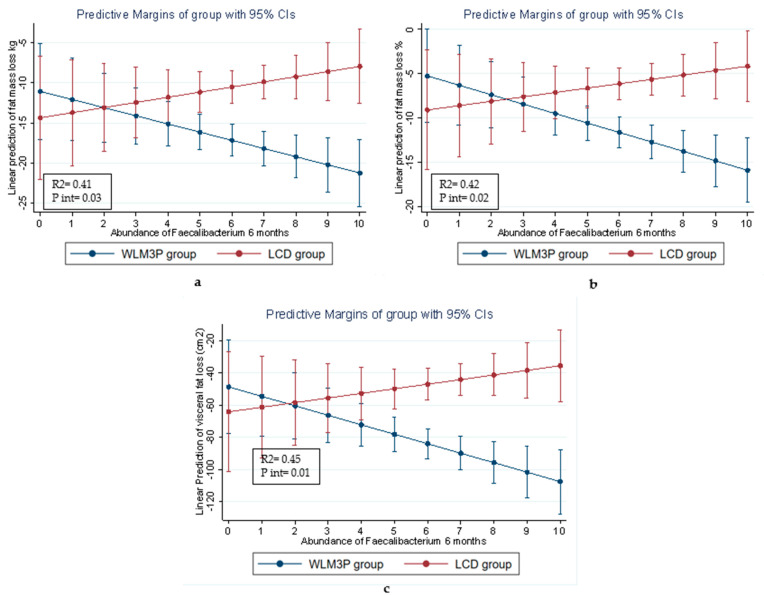
Predictive margins of the intervention groups (WLM3P and LCD) on weight-related outcomes as a function of the relative abundance of *Faecalibacterium* at 6 months. The graphs represent the linear prediction of (**a**) fat mass loss (kg), (**b**) fat mass loss (%), and (**c**) visceral fat loss (cm^2^) across a range of *Faecalibacterium* abundances in the gut microbiota normalized by Centered log ratio. In all three graphs, the blue line represents WLM3P group (blue line), and red line represents the LCD group. Error bars represent the 95% confidence intervals (CIs) for the predicted values, and the non-overlapping CIs in several cases suggest statistically significant differences between the two groups.

As shown in Figure 4, the abundance of *Faecalibacterium* at 6 months was significantly associated with reductions in fat mass (kg) (Figure 4a), fat mass percentage (Figure 4b), and visceral fat (Figure 4c), adjusted by age, sex and BMI. The WLM3P group displayed a strong, significant association between *Faecalibacterium* levels and reductions in these measures, while this relationship was weaker in the LCD group. These results suggest that the interaction between *Faecalibacterium* and the intervention (*p* < 0.001) was more pronounced in the WLM3P group, highlighting the potential role of this microbial genus in mediating the effects of diet on body composition in the context of the WLM3P intervention. Furthermore, while the predictive interaction between *Faecalibacterium* and total body weight loss (kg) and BMI reduction was also tested, these interactions were only marginally significant, with *p*-values of 0.10 and 0.08, respectively.

## 4. Discussion

This secondary analysis offers novel insights into how the multicomponent Weight Loss Maintenance 3 Phases Program (WLM3P) modulates gut microbiota over a 6-month active weight-loss phase in adults with obesity. Compared to a standard low-carbohydrate diet (LCD), the WLM3P group achieved significantly greater reductions in body weight, BMI, fat mass, visceral fat, and waist circumference, with no significant differences in muscle mass loss or blood lipid profiles. These results are consistent with prior large-scale findings from the WLM3P trial [22], although some discrepancies in lipid outcomes (e.g., HDL cholesterol, TG/HDL ratio) may reflect individual variability or a more limited scope of metabolic benefits.

The comprehensive design of WLM3P, incorporating caloric restriction, high-protein/low-carb macronutrient distribution, time-restricted eating (TRE), supplementation with fermentable fibers and polyphenols, and digital support, appears to drive more pronounced microbial remodeling than LCD alone, despite the observational nature of microbiota data.

Specifically, alpha diversity significantly increased and beta diversity was restructured in the WLM3P group, hallmarks of a more resilient and metabolically favorable gut ecosystem [5,6,12,13]. A key finding was the enrichment of *Faecalibacterium*, a butyrate-producing genus known for anti-inflammatory effects and gut barrier support. Its increased abundance aligns with previous studies on caloric restriction, TRE, and polyphenol-rich dietary patterns [14,20,26,27,28]. Mechanistically, *Faecalibacterium* may promote fat loss and metabolic improvement through SCFA production, gut hormone regulation (GLP-1, PYY), and AMPK activation [7,29]. In our study, higher *Faecalibacterium* in WLM3P group was associated with greater reductions in total and visceral fat (Figure 4), reinforcing its potential as a biomarker of diet responsiveness [5,7,9,10].

*Christensenellaceae R-7* also increased significantly in WLM3P, a taxon robustly associated with leanness, lower visceral fat, and improved metabolic profiles [11]. Similar shifts have been observed in response to caloric restriction, protein-rich diets, synbiotics, and intermittent fasting [8,30,31]. The presence of other enriched taxa, including *Phascolarctobacterium* and *Ruminococcaceae UCG_002/014*, further supports the establishment of a gut microbial profile conducive to metabolic regulation [14,32,33]. Conversely, the intervention reduced the relative abundance of pro-inflammatory and dysbiosis-associated taxa such as *Subdoligranulum*, *Streptococcus*, *Lachnospira*, and *Romboutsia*, all of which have been linked to impaired gut permeability and adverse metabolic profiles [13,34,35,36].

Correlation analyses support functional links between microbial shifts and metabolic outcomes. *Ruminococcaceae UCG_002* was negatively associated with waist-hip ratio, consistent with its role in regulating adiposity [32]. *Blautia* showed mixed correlations with lipid markers and muscle mass, reflecting its dual metabolic roles depending on host context [37,38]. *Phascolarctobacterium* is often linked to enhanced insulin sensitivity and anti-inflammatory activity [39]. However, its positive correlation with LDL and triglycerides in our study suggest context-specific effects (e.g., substrate availability or macronutrient influence), a finding mirrored in prior studies reporting both beneficial and lipogenic associations with this genus [33]. *Prevotella_2* correlated positively with triglycerides, despite evidence suggesting that certain *Prevotella* species contribute to improved lipid profiles via SCFA production [12,40], indicating that macronutrient context may shape its effects. In contrast, in the LCD group, *Akkermansia* negatively correlated with triglycerides, consistent with its lipid-lowering role [41], while *Romboutsia* was positively associated with BMI and fat mass, indicating a less favorable gut profile [34].

The observed microbial shifts in the WLM3P group are likely driven by the synergistic effects of the multiple dietary strategies integrated into the intervention.

Collectively, the integration of caloric restriction, tailored macronutrient distribution, TRE, and supplementation with fermentable fibers and polyphenol-rich compounds likely established a nutrient-rich gut environment alongside a temporally structured feeding pattern aligned with circadian rhythms. This combination may have enhanced gut barrier integrity, synchronized host–microbiota interactions and microbial diurnal oscillations, promoted saccharolytic fermentation, and supported the selective enrichment of SCFA-producing taxa such as *Faecalibacterium* and *Christensenellaceae*, while increasing overall microbial diversity [8,14,21,27,28,30,31]. These microbial shifts likely contributed to the clinically significant weight loss and reductions in adiposity observed in the WLM3P group.

While the WLM3P program yielded clinically significant results, it is important to acknowledge its intensive and multifaceted nature, which may limit immediate scalability in standard clinical settings. Nevertheless, the protocol offers a valuable framework for designing comprehensive obesity interventions that leverage the gut microbiota as a modifiable therapeutic target. These findings support the integration of microbiota-informed strategies into the personalization of dietary programs, with the goal of enhancing weight loss efficacy and improving metabolic health in a sustainable manner.

By advancing our understanding of these interactions, microbiota-targeted nutritional interventions could be developed to intentionally enhance beneficial taxa like *Faecalibacterium* and *Christensenellaceae R-7*, offering novel therapeutic strategies for obesity management.

Although microbiota remodeling was associated with improved adiposity, causal inference is not possible. Interestingly, these shifts occurred in the absence of major changes in blood lipids, suggesting that microbiota changes may precede systemic metabolic benefits. Monitoring the gut microbiome may thus offer early indicators of dietary intervention efficacy.

### Limitations

This study has several limitations. First, microbiota was only assessed after the 6-month weight loss phase, and therefore long-term microbial stability and clinical relevance during the weight maintenance period could not be evaluated. Second, the microbiota analysis was limited to a subset of participants, which reduced the overall sample size and may affect generalizability. Nonetheless, we applied robust statistical approaches, including non-parametric paired testing and FDR correction for multiple comparisons, to minimize the risk of false discoveries and ensure the reliability of the findings despite the modest cohort. However, comparisons showed no significant differences in baseline characteristics between included and non-included participants, supporting subgroup representativeness. Finally, the use of 16S rRNA sequencing restricts taxonomic resolution and functional insight; no metabolomic or functional profiling was performed.

Future studies should employ multi-omics platforms, combining metagenomics, metabolomics, and transcriptomics to explore microbial functionality and causal pathways. Long-term follow-up will also be critical to assess the durability of microbial shifts and their predictive value for sustained weight maintenance.

## 5. Conclusions

The WLM3P program resulted in significant improvements in anthropometric and body composition measures, alongside marked shifts in gut microbiota structure and diversity. Notably, the enrichment of SCFA-producing taxa such as *Faecalibacterium* and *Christensenellaceae R-7* highlights a potentially important microbiota-mediated component of the intervention’s metabolic benefits.

The observed association between *Faecalibacterium* abundance and reductions in fat mass and visceral adiposity suggests its value not only as a biomarker of dietary responsiveness but also as a possible therapeutic target. These findings reinforce the potential of gut microbiota modulation as a key target for individualized obesity management strategies, underscoring the need for translational research to bridge microbial shifts with clinically actionable outcomes.

Future studies employing functional metagenomics, metabolomics, and long-term follow-up are essential to establish causality, elucidate underlying mechanisms, and support the development of precision nutrition strategies tailored to individual microbiome profiles.

## Figures and Tables

**Table 1:** Comparative analysis of anthropometric and biochemical measurements in the WLM3P and LCD groups over time (baseline and 6 months).

Variables	WLM3P Group (CI)	LCD Group (CI)	Δ Groups (CI)	*p*-Value	Intervention Time
Weight (kg)					
Baseline	97.1 (93.3; 100.9)	96.4 (92.6; 100.2)	0.73 (−4.70; 6.15)	0.79	<0.001
6 months	76.5 (73.1; 80.8)	83.4 (79.6; 87.3)	−6.50 (−11.93; −1.07)	0.01	
Δ visit	−20.6 (−17.9; −22.4)	−12.9 (−10.7; −15.2)			
BMI (Kg/m^2^)					
Baseline	34.1 (33.3; 34.9)	34.2 (33.4; 34.9)	−0.12 (−1.20; −0.96)	0.82	<0.001
6 months	27.0 (26.3; 27.8)	29.6 (28.8; 30.4)	−2.61 (−3.69; −1.52)	<0.001	
Δ visit	−7.1 (−6.3; −7.8)	−4.6 (−3.9; −5.3)			
Fat mass (Kg)					
Baseline	41.5(39.1; 44.0)	41.1 (38.6; 43.5)	0.46 (−3.02; 3.94)	0.79	<0.001
6 months	24.5 (22.0; 26.9)	30.6 (28.1; 33.0)	−6.09 (−9.57; −2.61)	<0.001	
Δ visit	−17.0 (−14.9; −19.1)	−10.5 (−8.4; −12.5)			
Fat mass (%)					
Baseline	43.3 (40.7; 45.8)	42.9 (40.3; 45.5)			
6 months	31.7 (29.1; 34.3)	36.8 (34.2; 39.3)	0.39 (−3.25; 4.03)	0.79	<0.001
Δ visit	−11.6 (−9.7; −13.4)	−6.11 (−4.3; −7.9)	−5.06 (−8.70; −1.42)	0.07	
Skeletal muscle mass (kg)					
Baseline	30.9 (28.8; 33.0)	30.9 (28.8; 33.0)			
6 months	28.8 (26.8; 31.0)	29.3 (27.2; 31.4)	−0.03 (−3.00; 2.94)	0.98	0.14
Δ visit	−2.0 (1.6; −2.4)	−1.6 (1.2; −2.0)	−0.42 (−3.39; 2.56)	0.78	
Visceral fat (cm^2^)					
Baseline	200.3 (186.2; 213.8)	193.8 (180.3; 207.3)			
6 months	116.6 (103.1; 130.1)	147.3 (133.8; 160.8)	6.46 (−12.62; 25.55)	0.51	<0.001
Δ visit	−87.7(−73.9; −93.5)	−46.5 (−36.7; −56.3)	−30.70 (−49.78; −11.61)	<0.001	
Waist (cm)					
Baseline	100.3 (97.2; 103.4)	103.7 (100.6; 106.8)	−3.38 (−7.78; 1.02)		
6 months	83.6 (80.7; 87.0)	92.8 (89.6; 95.9)	−8.93 (−13.34; −4.52)	0.13	<0.001
Δ visit	−16.5 (−14.5; −18.4)	−10.9 (−8.9; −12.9)		<0.001	
Waist-to-hip ratio					
Baseline	1.04 (0.99; 1.04)	0.99 (0.97; 1.01)	0.03 (−0.01; 0.06)	0.11	<0.001
6 months	0.91 (0.88; 0.93)	0.94 (0.92; 0.96)	−0.04 (−0.07; −0.01)	0.02	
Δ visit	−0.1 (−0.1; −0.1)	−0.1 (−0.0; −0.1)			
LDL cholesterol (mg/dL)					
Baseline	114.0 (101.6; 126.5)	123.6 (111.2; 136.1)	−9.60 (−27.20; 8.00)	0.28	0.41
6 months	114.0 (101.5; 126.4)	118.3 (105.9; 130.8)	−4.37 (−21.97; 13.23)	0.62	
Δ visit	0.03 (−8.94; 9.00)	−5.27 (−14.24; 3.70)			
HDL cholesterol (mg/dL)					
Baseline	47.9 (43.6; 52.2)	52.4 (48.1; 56.6)	4.47 (10.51; 1.58)	0.15	0.16
6 months	56.3 (52.1; 60.6)	58.0 (53.7; 62.3)	1.70 (−4.34; 7.74)	0.58	
Δ visit	8.4 (11.14; 5.66)	5.6 (8.4; 2.9)			
Triglycerides (mg/dL)					
Baseline	129.1 (106.2; 151.9)	130.4 (107.5; 153.2)	−1.30 (−33.62; 31.02)	0.93	0.71
6 months	81.6 (58.8; 104.5)	89.3 (66.4; 112.1)	−7.63 (−39.96; 24.69)	0.64	
Δ visit	−47.4 (−71.6; 23.2)	−41.1 (−65.3; 16.9)			
Tg/HDL ratio					
Baseline	3.0 (2.3; 3.7)	2.7 (2.0; 3.4)	0.28 (−0.72; 1.28)	0.58	0.57
6 months	1.6 (0.9; 2.3)	1.6 (0.9; 2.4)	−0.03 (−1.03; 0.97)	0.95	
Δ visit	−1.4 (−2.2; 0.6)	−1.1 (−1.9; 0.3)			

**Table 2:** Bacterial taxa significantly different in WLM3P after 6 months (at genus level) analyzed by EdgeR.

Name	log2FC	*p*-Value	FDR
*Romboutsia*	−5.01	2.62 × 10^−8^	5.25 × 10^−7^
*Family XIII AD3011 group*	−4.29	2.60 × 10^−7^	2.63 × 10^−6^
*Subdoligranulum*	−4.33	5.85 × 10^−5^	3.90 × 10^−4^
*Phascolarctobacterium*	3.89	1.31 × 10^−4^	5.78 × 10^−4^
*Streptococcus*	−3.88	1.44 × 10^−4^	5.77 × 10^−4^
*Christensenellaceae_R_7_group*	2.66	6.92 × 10^−4^	0.002
*Ruminococcaceae UCG_005*	−3.46	0.002	0.006
*Agathobacter*	−2.28	0.005	0.011
*Ruminococcus 1*	2.58	0.005	0.011
*Dialister*	−2.87	0.009	0.019

Log2FC: logarithm 2-fold change (positive value when the abundance increases after 6 months of the intervention); FDR: False Discovery Rate.

**Table 3:** Bacterial taxa significantly different in LCD group after 6 months (at genus level) analyzed by EdgeR.

Name	log2FC	*p*-Value	FDR
*Bacteroides*	38.234	1.03 × 10^−4^	0.001
*Lachnospira*	28.216	4.57 × 10^−4^	0.003
*Phascolarctobacterium*	29.346	5.42 × 10^−4^	0.003
*Ruminococcaceae_UCG_014*	27.338	0.003	0.013
*Ruminococcaceae_UCG_005*	27.072	0.005	0.017
*Ruminococcaceae_UCG_002*	1.984	0.002	0.048

Log2FC: logarithm 2 fold change (positive value when the abundance increases after 6 months of the intervention); FDR: False Discovery Rate.

## Data Availability

The full data sets generated during and/or analyzed during the current study are not publicly available because the ethics committee only allowed the use of the data in the context of the present research project; however, anonymized partial data sets or summaries of the data are available from the corresponding author on reasonable request.
